# The Synergetic Effect of Egyptian *Portulaca oleracea* L. (Purslane) and *Cichorium intybus* L. (Chicory) Extracts against Glucocorticoid-Induced Testicular Toxicity in Rats through Attenuation of Oxidative Reactions and Autophagy

**DOI:** 10.3390/antiox11071272

**Published:** 2022-06-27

**Authors:** Samar R. Saleh, Ashraf Manaa, Eman Sheta, Doaa A. Ghareeb, Nihad M. Abd-Elmonem

**Affiliations:** 1 Bio-Screening and Preclinical Trial Lab, Biochemistry Department, Faculty of Science, Alexandria University, Alexandria 21515, Egypt; ashraf.manaa@alexu.edu.eg (A.M.); d.ghareeb@alexu.edu.eg (D.A.G.); nehad.abdelgwad@alexu.edu.eg (N.M.A.-E.); 2 Department of Pathology, Faculty of Medicine, Alexandria University, Alexandria 21131, Egypt; iman.sheta@alexmed.edu.eg

**Keywords:** oxidative stress, insulin resistance, endoplasmic reticulum stress, Sigma 1R and GRP78, LC3II, P62, Phospho-mTOR (Ser2448), α-mannosidase activity, combination index analysis

## Abstract

Long-term glucocorticoids can alter sperm motility, vitality, or morphology, disrupting male reproductive function. This study scrutinized the synergistic benefits of two Egyptian plants against dexamethasone (Dexa)-induced testicular and autophagy dysfunction in male rats. Phytochemical ingredients and the combination index were estimated for Purslane ethanolic extract (PEE) and Chicory water extract (CWE). Four control groups received saline and 100 mg/kg of each PEE, CWE, and PEE/CWE, daily for 8 weeks. Dexa (1 mg/kg daily for 6 weeks) induced infertility where PEE, CWE, and PEE/CWE were given. Seminal analysis, male hormones, glycemic and oxidative stress markers, endoplasmic reticulum (ER) stress markers (Sigma 1R and GRP78), and autophagy regulators (Phospho-mTOR, LC3I/II, PI3KC3, and Beclin-1, P62, ATG5, and ATG7) were measured. The in vitro study illustrated the synergistic (CI < 1) antioxidant capacity of the PEE/CWE combination. Dexa exerts testicular damage by inducing oxidative reactions, a marked reduction in serum testosterone, TSH and LH levels, insulin resistance, ER stress, and autophagy. In contrast, the PEE and CWE extracts improve fertility hormones, sperm motility, and testicular histological alterations through attenuating oxidative stress and autophagy, with a synergistic effect upon combination. In conclusion, the administration of PEE/CWE has promised ameliorative impacts on male infertility and can delay disease progression.

## 1. Introduction

Infertility and problems of impaired fecundity have been considered the most serious social problems facing developed and industrialized countries. Infertility has been defined as the failure to achieve conception after 12 months or more of regular unprotected sexual intercourse [1,2]. In recent decades, infertility has impacted an increasing number of couples. Infertility affects 8–12% of couples worldwide, accounting for 48.5 million couples globally, and is one of the primary reasons for divorce [3]. Of all infertility cases, “male factor” infertility accounts for approximately half of couple infertility (40–50% of instances), and as many as 2% of all men will exhibit suboptimal sperm parameters. It may be due to low sperm concentration (oligozoospermia), poor sperm motility (asthenozoospermia), abnormal sperm morphology (teratozoospermia), or a combination of these [2,4]. Although the etiology of infertility in up to 60% of cases remains unclear and is referred to as idiopathic [5]. Several etiological factors have been identified in infertile men. Lifestyle and humans’ exposure to free radicals, environmental pollutants, and drug treatment could adversely affect the male reproductive system [4,6]. Glucocorticoids (GCs) are the major steroid hormones secreted by the adrenal gland. Prolonged treatment with GCs was found to cause side effects that can cause endocrine abnormalities via interfering with the anterior hypothalamic–pituitary–gonadal (HPG) axis [7,8]. Dexamethasone (Dexa) is a synthetic glucocorticoid widely used in therapy due to its immunosuppressive and anti-inflammatory effects [9]. In recent years, Dexa has been frequently prescribed [10]. Low doses of Dexa were found to decrease insulin sensitivity, resulting in hyperinsulinemia and impaired glucose tolerance, increasing the risk of insulin resistance and subsequently developing type 2 diabetes [11,12,13]. Dexa was found to induce testicular deficiency and impair the male reproductive functions. Moreover, Dexa has adverse effects on testicular tissue via affecting the gonadotropic axis and thus suppressing testosterone production. Dexa also affects the testes and spermatogenesis indexes by reducing daily sperm production and disrupting sperm motility [10,14]. In addition, Dexa induces the overproduction of reactive oxygen species (ROS), causing oxidative stress and dysregulation of physiological processes in many tissues and organs, including the testicular germ cells [15,16]

Oxidative stress is among the core causes of male infertility [17,18]. During oxidative stress, the endogenous testicular antioxidants are inadequate to protect the developing germ cells from excess ROS. Normal physiological levels of ROS are crucial for male reproductive functions and spermatogenesis. However, the supraphysiological concentration of ROS can be responsible for oxidative damage to the intracellular components, proteins, lipids, and nucleic acids (DNA, RNA), resulting in impaired sperm parameters, testicular injury, and, eventually, male infertility [18,19,20]. Moreover, oxidative stress can disrupt the protein-folding mechanism, resulting in the accumulation of unfolded or misfolded proteins, causing further endoplasmic reticulum (ER) stress [21,22].

Furthermore, ER stress and oxidative stress play significant roles in the pathophysiology of male infertility by inducing autophagy in testes, causing testicular degeneration and dysfunction [22,23]. Autophagy is a conserved process of cellular homeostasis. Autophagy plays an important role in cell survival and maintenance by degrading and recycling dysfunctional and unnecessary cellular components, protein aggregates, and macromolecules, to provide energy and essential materials. Interestingly, autophagy is involved in a wide range of cellular events within the male reproductive system. In spermatogenesis, proper autophagy is mandatory for the formation of specific structures that guarantee correct early and late spermatogenesis [24]. However, cumulative studies reveal that autophagy is a double-edged sword [25]. Autophagy disorder may also promote cytopathy, decreased serum testosterone, and cause sperm differentiation defects [26]. Thus, oxidative stress-induced autophagy is considered one of the proposed mechanisms for idiopathic male infertility [27].

According to previous assumptions, the Mediterranean diet and different natural remedies rich in flavonoids and antioxidants can promote the elimination of pollutants and hinder oxidative stress and testicular damage through different mechanisms [28,29,30,31]. Purslane (*Portulaca oleracea* L.) and Chicory (*Cichorium intybus* L.) are edible Egyptian plants that belong to the family Portulacaceae and Asteraceae, respectively [32,33,34]. *P. oleracea* leaves are a rich source of dietary antioxidants, including flavonoids (flavonol glycosides, such as kaempferol and quercetin), glutathione, omega-3 fatty acids, alkaloids, and vitamins, as well as dietary minerals [35]. Phytochemical analysis of Chicory root indicated that it contains inulin, alkaloids, flavonoids, coumarins, sesquiterpene lactones, vitamins, minerals, and volatile oils [36]. The secondary metabolites found in Purslane and Chicory have been shown to have potent antioxidant, metal chelating, and anti-inflammatory activities that positively impact human and livestock health [37,38]. The role of Chicory in increasing testosterone, fructose levels, mannosidase activity, and sperm mortality and vitality has been reported [39,40]. Both Purslane and Chicory extracts’ neutralizing free radicals’ effects can prevent the adverse effect of ROS on spermatogenesis, protect the sperm, and improve the male reproductive function.

Based on these premises, the present study was designed to investigate the synergetic protective effects of a combination of Purslane and Chicory extracts against Dexa-induced testicular damages by studying the changes in the testicular morphology, spermatogenic indices, and biochemical parameters in adult rats. Collectively, we elaborate the role of Dexa-induced oxidative stress in the aggravation of ER stress and autophagy signaling via exploring the levels of the glycemic markers, ER stress regulators (Sigma 1R and glucose-regulated protein 78 (GRP78)), and autophagy regulators responsible for autophagosome initiations (P-mTOR(S2448), beclin-1, phosphatidylinositol 3-kinase class III (PI3KC3)), formation (autophagy genes ATG5 and ATG7), and maturation (LC3I/II, P62) in the rat’s testis.

## 2. Materials and Methods

### 2.1. Chemicals

Folin–Ciocalteau reagent, aluminum chloride, nitroblue tetrazolium (NBT), 1-1-diphenyl 2-picrylhydrazyl (DPPH), phenazine methosulfate (PMS), nicotinamide adenine dinucleotide reduced (NADH), gallic acid, catechin, thiobarbituric acid, trichloroacetic acid (TCA), Cumene H_2_O_2_, Tris-HCl, 5,5′-dithio-bis-2-nitrobenzoic acid (DTNB), reduced glutathione (GSH), Griess reagent (Sulphanilamide and *N*-1-naphthyl ethylenediamine), sulfosalicylic acid, and dexamethasone (Dexa) were obtained from Sigma-Aldrich (St. Louis, MO, USA). ELISA kits were purchased from COBAS, USA. Other assay kits were purchased from Spectrum, Egypt. Easy red^TM^ total RNA extraction kit, cDNA synthesis kit, and 2X SYBR green master mix kit were obtained from iNtRON Biotechnology, Korea. Primers were purchased from Invitrogen, Thermo Fisher Scientific, Waltham, MA, USA. The following antibodies were used: Anti-beclin-1 (PRS3613, Sigma-Aldrich, Merck KGaA, Darmstadt, Germany), phosphoinositide-3-kinase class 3 (Anti-PI3KC3, abx329840, Abbexa Ltd., Cambridge, UK), Anti-Sigma R1 polyclonal antibody (abx241819, Abbexa Ltd., Cambridge, UK), Phospho-mTOR (Ser2448) (2971, Cell signaling technology), Anti-P62/SQSTM1 (D1Q5S) rabbit mAb (39749, Cell Signaling Technology, Danvers, MA, USA), LC3B antibody-N-terminal (ab229327, Abcam, Cambridge, UK), glucose-regulated protein 78 (GRP78) polyclonal antibody (Invitrogen, Thermo Fisher Scientific Inc., Waltham, MA, USA, PAS-85169), and β-actin (antibody, Genie, Redmond, WA, USA, CABC026). Other chemicals were obtained with high grades.

### 2.2. Plant Materials

**Figure 1:**
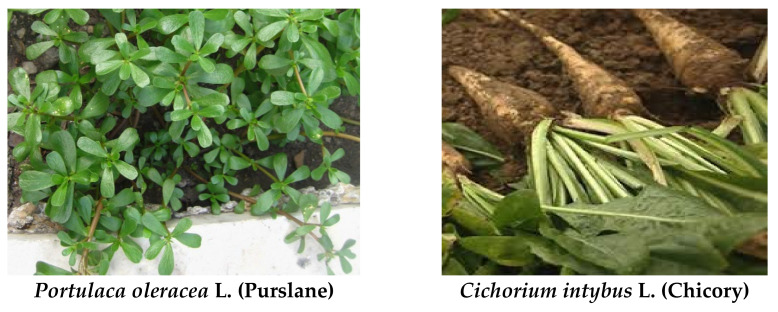
Illustrated *Portulaca oleracea* L. (Purslane) and *Cichorium intybus* L. (Chicory) plants.

*Portulaca oleracea* L. (Purslane) leaves and *Cichorium intybus* L. (Chicory) roots (Figure 1) were collected from Egyptian farmers (El Behera, Egypt) during winter 2019 and recognized by a scientist in the Botany Department, Faculty of Science, Alexandria University.

### 2.3. Preparation of the P. oleracea and C. intybus Extracts

#### 2.3.1. Preparation of *P. oleracea* (Purslane) Ethanolic Extract (PEE)

Fresh samples were collected, cleaned with water, and were split as roots, stems, and leaves. Leaves were crushed into powder by a plant grinder after being dried by sun exposure at room temperature [41]. The obtained powder (600 g obtained from about 10 kg fresh leaves) was placed in a sealed vessel mixed with 5 its volume of ethanol (70%). The mixture was filtrated, reduced using a rotary evaporator, then lyophilized, and the final powder was ready to be used (PEE, yield of 15%).

#### 2.3.2. Preparation of *C. Intybus* (Chicory) Water Extract (CWE)

Apparently, healthy Chicory roots were collected, washed thoroughly in tap water, dried at room temperature, and then lyophilized at −80 °C to be powdered. Dried powdered root (500 g) was soaked in 500 mL water for 4 days. The extract was decanted, filtered through cheesecloth, and was reduced to 10% of its original volume [34], then lyophilized to get the final powder (CWE) that was stored at −20 °C until used (CWE, yield of 9%).

### 2.4. The In Vitro Study

#### 2.4.1. Phytochemical Contents, Mineral Analysis, and Vitamins

**The total phenolic content** of PEE and CWE was determined using the Folin–Ciocalteau colorimetric method [42]. The resulted blue color was measured at 760 nm against a blank reagent, where gallic acid was employed as standard. **Total flavonoid content** was quantified calorimetrically using the aluminum chloride method, and catechin was used as standard [43]. Data are expressed as mg equivalent/g extract.

**The mineral content** (Ca, Cu, Fe, K, Mg, Mn, Na, P, Se, and Zn) of PEE or CWE was assessed. One gram of PEE and CWE was digested in a hydrochloric acid/nitric acid mixture (3:1) for 1 h at 100 °C. The digested extracts were proceeded to be available for the analysis using atomic absorption spectroscopy (Agilent 5100 SVDV ICP-OES, US) according to the US EPA Method 200.7 [44,45] and US EPA Method 6010C [46]. Data are expressed as µg/g extract.

**Vitamins content:** Folic acid, vitamin C, and vitamin A were analyzed in the Laboratory Unit for Advanced Environmental and Biological Analysis, High Institute of Public Health, Alexandria University. Folic acid was extracted from PEE and CWE according to the method described in AOAC [47]. The HPLC technique determined the folic acid content using standard folic acid (100 μg/mL). Vitamin C was extracted in a mixture of meta-phosphoric acid (0.3 M) and acetic acid (1.4 M) (1:1 *w/v* ratio) according to the modified method of Abushita, Hebshi [48]. The mixture was agitated at 100 rpm for 15 min at room temperature and then filtered to obtain a clear extract. Vitamin C content was quantified using a reverse-phase HPLC technique with standard vitamin C (1 mg/mL) according to the method of Ismail and Cheah [49]. As a fat-soluble vitamin, vitamin A was extracted in isopropanol and determined by HPLC using retinol acetate as a standard, following Kozlov, Solunina [50].

#### 2.4.2. HPLC Analysis for Phenolic Compounds Profile

Phytochemical components of PEE and CWE were identified and quantified by an Agilent 1260 Infinity HPLC series (Agilent Technologies, Santa Clara, CA, USA), as described by Lu, Yuan [51]. HPLC analysis was carried out at the Food Safety and Quality Control Laboratory (FSQC 0911-0915/2019), Faculty of Agriculture, Cairo University, Egypt. Briefly, 20 μL samples were separated on a Kinetex EVO C18 column (100 mm × 4.6 mm). The elution was performed using 0.2% H_3_PO_4_, methanol, and acetonitrile. The device was equipped with a variable-wavelength detector set at 284 nm. Retention time and peak spectra of the standard phenolic compounds—ran at the same chromatographic conditions—were used for identification. All phenolic compounds were expressed as µg/g extract.

#### 2.4.3. In Vitro Antioxidant Activities of PEE, CWE and Their Combination

For the antioxidant assays, the extracts were dissolved in dis. water (10 mg/mL) and concentration-dependent dilutions were made. The radical scavenging activity of the extracts was evaluated using 1-1-diphenyl 2-picrylhydrazyl (DPPH), nitric oxide (NO), and superoxide radical scavenging methods, as well as total antioxidant capacity.

**DPPH scavenging activity:** According to Manzocco, Anese [52], PEE and CWE (100 µL) were diluted, and 100 µL of DPPH solution (0.1 mM in methanol) was added. After 30 min dark incubation, the absorbance was measured at 517 nm [53].

**The superoxide anion scavenging activity:** The superoxide anion radicals were generated by NADH oxidation and assayed by a reduction in NBT. The reaction mixture (3.0 mL) contained 1.0 mL extract (of different concentrations), 0.5 mL of NBT (0.3 mM), 0.5 mL NADH (0.936 mM) and 0.5 mL Tris–HCl buffer (16 mM, pH 8.0). The reaction was started by the addition of 0.5 mL PMS solution (0.12 mM) to the reaction mixture and incubated at 25 °C for 5 min. The absorbance was measured at 560 nm against a blank sample [54].

**Nitric oxide scavenging activity:** According to Marcocci, Maguire [55], 100 µL sodium nitroprusside (10 mM in phosphate buffer, 7.4) was mixed with 50 µL of different concentrations (1–10 mg/mL) of PEE and CWE. The mixture was incubated at 25 °C for 180 min. Afterward, 150 µL of Griess reagent (1% sulfanilic acid and 1% naphthylethylenediamine dichloride (NED) dissolved in 2.5% phosphoric acid and mixed immediately before use) was added, and the mixture was incubated at room temperature for 30 min. The absorbance was measured at 546 nm.

The percentage of the radicals scavenging was calculated as follows:

$$\%\;\mathrm{inhibition}\;=\frac{A_{\mathrm{control}}\;-\;A_{\mathrm{sample}}}{A_{\mathrm{control}}}\;\times \;100$$

where A_sample_ is the absorbance of the extracts and A_control_ is the absorbance of the control (with no extract).

IC50 values were calculated to determine the 50% inhibition of the radicals. IC50 of both PEE and CWE for each assay was estimated, and the values were used for the mixture (IC50 PEE/IC50 CWE). Different ratios were used (1:1, 1:2, 1:4, 4:1) to estimate the antioxidant activities. A 1:1 (IC50 PEE: IC50 CWE) ratio showed the most synergetic effect; so, it was used in the in vivo study for the combined extracts.

**Total antioxidant capacity** was carried out using the phosphomolybdenum method [56]. An aliquot of the PEE and CWE solutions (100 µL, 2 mg/mL) was mixed with 1.9 mL of the reagent solution (0.6 M sulfuric acid, 28 mM sodium phosphate, and 4 mM ammonium molybdate). The test tubes were covered and incubated at 95 °C for 90 min. After that, the samples were cooled, and the absorbance was measured at 795 nm. Ascorbic acid was used as standard. The antioxidant capacity was estimated as mg ascorbic acid eq/g extract.

### 2.5. Experimental Design

#### 2.5.1. Animals

Sixty-four sexually mature Sprague-Dawley male rats, weighing about 165–180 g (10–12 weeks), were purchased from the animal house of the Institute of Graduate Studies and Research, Alexandria University, Egypt. All rats were raised in polypropylene cages (4 animals/cage) and fed on a standard diet with ad libitum tap water. They were kept on a 12:12 h light-dark cycle in a well-aerated room under a controlled environment. All animal procedures were accepted under the ethical standards of scientific research of the Institutional Animal Care and Use Committee (IACUC), Alexandria University (AU: 04 19 10 21 1 02, approved on 26 October 2019).

#### 2.5.2. Induction of Male Infertility and Treatments

After one week of acclimatization, rats were divided into eight groups (8 animals/group) and treated for 8 weeks. There are four control groups that received saline (Sham), PEE (100 mg/kg), CWE (100 mg/kg), and PEE/CWE combined (100 mg/kg), daily for eight weeks. Infertility-induced group: received dexamethasone (Dexa, 1 mg/kg, sc, dissolved in saline) daily for six weeks. There are three treated groups that received: PEE (Dexa + PEE, 100 mg/kg), CWE (Dexa + CWE, 100 mg/kg), and PEE/CWE combined (Dexa + PEE/CWE, 1/4, 20/80 mg/kg). The animals of the treated groups were continuously administered their respective treatments via gavage daily for two weeks before Dexa and continued for another six weeks with Dexa. Based on earlier studies, a daily dose of 100 mg/kg of either PEE or CWE was chosen [40,57,58].

#### 2.5.3. Blood and Testicular Tissues Collection

At the end of the experimental period, the rats were fasted overnight and sacrificed by decapitation. Blood samples were collected from the inferior vena cava. Sera were separated by centrifugation and stored at −20 °C for glycemic markers and hormone measurements.

Testicles from each animal were dissected. For histopathological examination, four right testes/group were fixed in 10% neutral formalin. The other four right testes/group were homogenized for the seminal analysis assessment. The left testis was kept at −80 °C for biochemical and molecular measurements. The testicular tissue was crushed in Tris HCl buffer (20 mM, pH 7.4) (1:9, *w*/*v*). The supernatant was kept at −80 °C until used for enzymatic and non-enzymatic analysis (fructose level, α-mannosidase activity, and oxidative stress markers).

### 2.6. Testicular Seminal and Biochemical Analysis

#### 2.6.1. Seminal Analysis

Testes samples were quickly removed. One testis was manually opened with scissors to release spermatozoa in a small clean petri dish containing 2 mL of Ham’s F-10 containing 0.5% bovine serum albumin (BSA) at 37 °C. Testicular sperm count, morphology index, and motility were assessed manually using a hemocytometer. All procedures were carried out at 37 °C, and everything that came in contact with sperm was pre-warmed and kept at 37 °C. Each sample was counted four times and averaged [7,59].

#### 2.6.2. Hormone Measurements

Serum follicle-stimulating hormone (FSH), luteinizing hormone (LH), and testosterone levels were evaluated using ELISA kits (COBAS, Indianapolis, IN 46256, USA) according to the manufacturer’s instructions. The absorbance was measured at 450 nm (ELISA reader, ELx800, Bio-TEK, Winooski, VT, USA), and their levels were expressed as μIU/mL (FSH and LH) and ng/dl (testosterone).

#### 2.6.3. Fructose Levels and α-Mannosidase Activity

**Serum and testicular fructose levels** (sperm energy source) were determined according to Foreman, Mongar [60]. A total of 500 μL of either serum, testis homogenate supernatant, standard fructose (300 mg/dL), or distilled water (blank) was mixed with 500 µL TCA (1 mol/L), left for 10 min at room temperature, and then centrifuged at 3000 rpm for 10 min. A total of 50 µL of the supernatant was added to 100 µL resorcinol (9 mmol/L) and 1 mL hydrochloric acid (9 mol/L). The solution was placed in a boiling water bath for 5 min, and the developed color was measured at 495 nm against the blank using a spectrophotometer. Fructose levels were calculated and expressed as mg/dL or mg/g tissue in serum and testicular tissue, respectively.

**Testicular α-mannosidase** (EC 3.2.1.24) activity was assayed as described by Tulsiani, Skudlarek [61]. Briefly, 20 µL of 4-nitrophenyl-α-d-mannopyranoside (4 mM) and 20 µL of the testis homogenate supernatant were added to 100 µL of sodium acetate buffer, pH 4.4, and incubated for 60 min at 37 °C. The reaction was stopped by adding 100 µL of alkaline buffer (0.133 M glycine, 0.067 M NaCl, and 0.083 M Na_2_CO_3_ adjusted to pH 10.7 with 0.2 M NaOH). The p-nitrophenol released was quantized by measuring the absorbance of the sample against the blank at 400 nm. One unit of the PNP-α-mannosidase activity is the enzyme that catalyzes the release of 1 µmol p-nitrophenol per hour. The α-mannosidase activity was expressed as U/mg protein.

#### 2.6.4. Glycemic Markers

Serum insulin and fasting blood glucose (FBG) levels were measured using a commercial kit obtained from COBAS, Germany, and Spectrum-diagnostics, Egypt, respectively. Insulin sensitivity was estimated by homeostasis model assessment (HOMA-IR), and the β-cell function was evaluated by HOMA-β according to the following equations [62]:

$$\mathrm{HOMA}-\mathrm{IR}=\frac{\mathrm{Fasting}\;\mathrm{glucose}\;\left(\mathrm{mg}/\mathrm{dL}\right)\times \;\mathrm{Fasting}\;\mathrm{insulin}\;\left(\mu U/\mathrm{mL}\right)}{405}$$

$$\mathrm{HOMA}-\beta =\frac{360\times \;\mathrm{Fasting}\;\mathrm{insulin}\;\left(\mu U/\mathrm{mL}\right)}{\left[\mathrm{fasting}\;\mathrm{glucose}\;\left(\mathrm{mg}/\mathrm{dL}\right)-63\right]}$$

#### 2.6.5. Oxidative Stress Markers

Lipid peroxidation was assessed calorimetrically in testicular tissues by measuring the formation of **malondialdehyde (MDA)**, one of the aldehyde products of lipid peroxidation. MDA reacts with thiobarbituric acid under acidic conditions forming a pink-colored product on heating, measured spectrophotometrically at 532 nm [63]. The testicular MDA level was expressed as µmol/mg protein.

**The testicular reduced glutathione (GSH)** level was measured in the clear supernatant using Ellman’s method. After protein removal, GSH interacts with Ellman reagent (5,5′-dithio-bis-2-nitrobenzoic acid, DTNB), generating a yellow-colored 2-nitro-5-thiobenzoic acid product. The reaction between GSH and DTNB was analyzed at 412 nm. The GSH level was expressed as mM/mg protein.

The activity of **testicular glutathione peroxidase (GPx)** (EC 1.11.1.9) was determined based on the reduction of cumene hydroperoxide via GSH oxidation into glutathione disulfide (GSSG) [64]. The amount of utilized cumene hydroperoxide is determined directly by estimating the GSH content using DTNB. The GPx activity was determined by subtracting the excess GSH after enzymatic reaction from the total GSH in the absence of enzyme. The absorbance was read at 412 nm, and GPx activity was expressed as U/mg protein.

**The testicular glutathione-S-transferase (GST)** (EC 2.5.1.18) activity was spectrophotometrically determined using 4-nitrobenzyl chloride (pH 6.5) and GSH as substrates forming glutathione nitrobenzyl [65]. The absorbance was read at 310 nm, and GST activity was expressed as U/mg protein.

**Total testicular proteins** were determined using Lowry method, where BSA (1 mg/mL) was used as a standard [66]. Total protein content was used to calculate the antioxidant enzymes’ specific activity (unit activity/mg protein).

#### 2.6.6. ER Stress and Autophagy Regulators by ELISA Technique

The protein levels of testicular Sigma 1 receptor (Sigma 1R), phosphatidylinositol 3-kinase Class III (PI3KC3), and Beclin-1 were measured by the quantitative ELISA technique using rabbit polyclonal sigma 1R (#abx241819, Abbexa Ltd., Cambridge, UK), PI3KC3 (#abx329840, Abbexa Ltd., Cambridge, UK) and Beclin-1 (#PRS3613, Sigma-Aldrich, Merck KGaA, Darmstadt, Germany, USA). The antigen was diluted in a coating buffer (carbonate buffer, 0.2 M, pH 9.6) to a final concentration of 100 µg protein. In duplicate, samples were loaded in microtiter plate wells and incubated for 2 h at room temperature and overnight at 4 °C. A blocking solution of 5% BSA was utilized and incubated for 1 h at room temperature. To decrease the non-specific binding, primary and secondary antibodies were diluted in a blocking solution (2%). The primary antibodies were incubated for 1 h at room temperature before being kept at 4 °C overnight. The plate was washed six times with washing buffer before being incubated for 2 h at room temperature with alkaline phosphatase-conjugated secondary antibody (Goat anti-rabbit IgG, ALP, #A8025, Sigma-Aldrich, Merck KGaA, Darmstadt, Germany). ρ-Nitrophenyl phosphate disodium salt was used as ALP substrate and allowed for 15 min before the addition of sodium hydroxide (3 M) was used to stop the reaction. The developed color was measured at 450 nm on a plate reader. Their levels were expressed in ng/mg protein using the proteins’ standard curves.

### 2.7. Molecular Analyses

#### 2.7.1. Quantitative Real-Time Reverse Transcription PCR Analysis (qRT-PCR)

Total RNA from the testis was extracted according to the manufacturer’s instructions using an easy red^TM^ total RNA extraction kit (iNtRON Biotechnology, Gyeonggi-do, Korea) and measured spectrophotometrically. Then, 1 µg of total RNA was reverse transcribed using Maxime RT PreMix kit (iNtRON Biotechnology, Seongnam-si, Korea) following the manufacturer’s protocol. The qRT-PCR analysis was performed as follows: 1 µL of cDNA was added to appropriate primers (10 µM), 10 µL of RealMOD™ Green w2 2X qPCR mix (iNtRON Biotechnology, Korea), and RNase-free water in a final volume of 20 µL per well. Quantitative PCR was performed in Roto Gene Q 5Plex HRM (Qiagen, Hilden, Germany), applying the following thermal protocol: 95 °C for 2 min, followed by 35–40 cycles of 95 °C for 20 s, 43–60 °C for 30 s, and 72 °C for 60 s. Samples were loaded in duplicate. The comparative 2^−ΔΔCT^ method was used to calculate the data, and all values were normalized to the GAPDH gene’s mRNA level. The results are expressed as an *n*-fold increase in gene expression using the calibrator results of the sham control. Table 1 summarizes the primer sequences of the targeted genes and qRT-PCR conditions.

#### 2.7.2. Western Blot Analysis

Testicular tissue was homogenized in ice-cold Nonidet-P40 (NP-40) buffer with the addition of 1% protease inhibitor and phosphatase inhibitor cocktail. The lysates supernatant was separated by cool centrifugation at 13,000 rpm for 20 min, and their protein content was measured spectrophotometrically, followed by boiling in 2× Laemmli buffer for protein denaturation. Equal pooled protein concentrations (40μg) were loaded into 12% SDS-PAGE for separation and transferred onto nitrocellulose membranes (#1620094, Bio-Rad, Hercules, CA, USA). Membranes were blocked in 1× Tris-buffered saline/0.1% Tween 20 (TBST) with 5% BSA for 1 h at room temperature and incubated with the primary antibody specific to LC3B (ab229327, Abcam, Cambridge, UK), Phospho-mTOR(Ser2448) (2971, Cell signaling technology, Danvers, MA, USA), P62/SQSTM1 (39749, Cell Signaling Technology), GRP78 polyclonal antibody (PAS-85169, Invitrogen, Thermo Fisher Scientific, Waltham, MA, USA) and β-actin (antibody, Genie, Redmond, WA, CABC026) for 1 h at room temperature then overnight at 4 °C. The membrane was washed six times (10 min each) in 1× TBST and incubated with goat anti-rabbit alkaline phosphatase-conjugated secondary antibody for 2 h at room temperature. Then nitro blue tetrazolium/5-Bromo-4-chloro-3-indolyl-phosphate (NBT/BCIP) solution (Thermo Fisher Scientific, Waltham, MA, USA) was added to visualize the protein bands. Bands were measured with Image Studio Lite software (LI-COR Biotechnology, Lincoln, NE, USA). All expressed proteins were normalized to the β-actin content, and their relative expression was calculated as the fold change of control.

### 2.8. Combination Index Analysis

The nature of the combined effects of PEE and CWE was determined using the method described by Zhou, Li [71]. The combination of extracts may significantly provide better (synergistic), worse (antagonistic), or no new (additive) outcome compared to the single extract. This new effect can be investigated by calculating the combination index (CI) that quantifies the synergism or antagonism [72]. For the in vitro antioxidant parameters, the predictive value for the PEE/CWE combination was calculated using the following formula:

$$\mathrm{The}\;\mathrm{Predicted}\;\mathrm{value}=\left[\frac{\mathrm{IC}50\;\mathrm{PEE}}{2}+\frac{\mathrm{IC}50\;\mathrm{CWE}}{2}\right]$$

While for other in vivo tested parameters,

$$\mathrm{The}\;\mathrm{Predicted}\;\mathrm{value}\;=\left[\frac{\mathrm{Observed}\;\mathrm{value}\;\mathrm{for}\;\mathrm{PEE}}{\mathrm{Control}\;\mathrm{value}}+\frac{\mathrm{Observed}\;\mathrm{value}\;\mathrm{for}\;\mathrm{CWE}}{\mathrm{Control}\;\mathrm{value}}\right]\times Control\;value$$

The CI was calculated using the formula:

$$\mathrm{CI}=\frac{\mathrm{Obseved}\;\mathrm{value}}{\mathrm{Predicted}\;\mathrm{value}\;\mathrm{of}\;\mathrm{combination}}$$

A CI of less than, equal to, or more than 1 indicates a synergic, additive, or antagonistic effect, respectively [71].

### 2.9. Histopathological Study

Testicular tissues were removed from different rat groups after scarification. They were fixed in 10% neutral formalin and processed into paraffin blocks. Hematoxylin and eosin (H&E)-stained, four-microns-thick sections were mounted on glass slides [73]. They were examined by a pathologist using Olympus CX22 light microscopy. Spermatogenesis was assessed in seminiferous tubules by measuring the means of the spermiogenesis index (SI) in testicular tissues. It was evaluated by calculating the percentage of tubules containing mature sperms using image analysis software (Leica application suite software, version 4.12.0). At least 200 tubules were assessed in each section [74]. In addition, a morphologic assessment of the tubular size and lining cells was done.

### 2.10. Statistical Analysis

The values were presented as the mean ± SD. Differences within groups were statistically examined using one-way ANOVA using the LSD test, with a *p* < 0.05 regarded as statistically significant. SPSS 16.0 (Chicago, IL, USA) was utilized for these studies.

## 3. Results

### 3.1. Characterization of PEE and CWE

**Figure 2:**
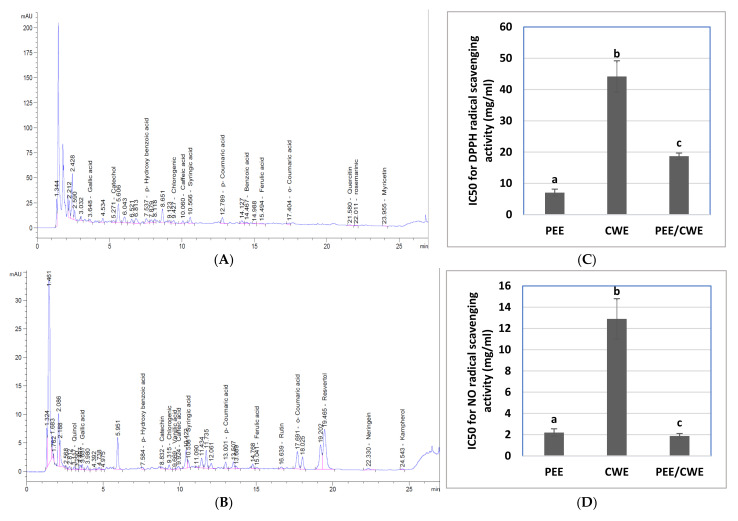

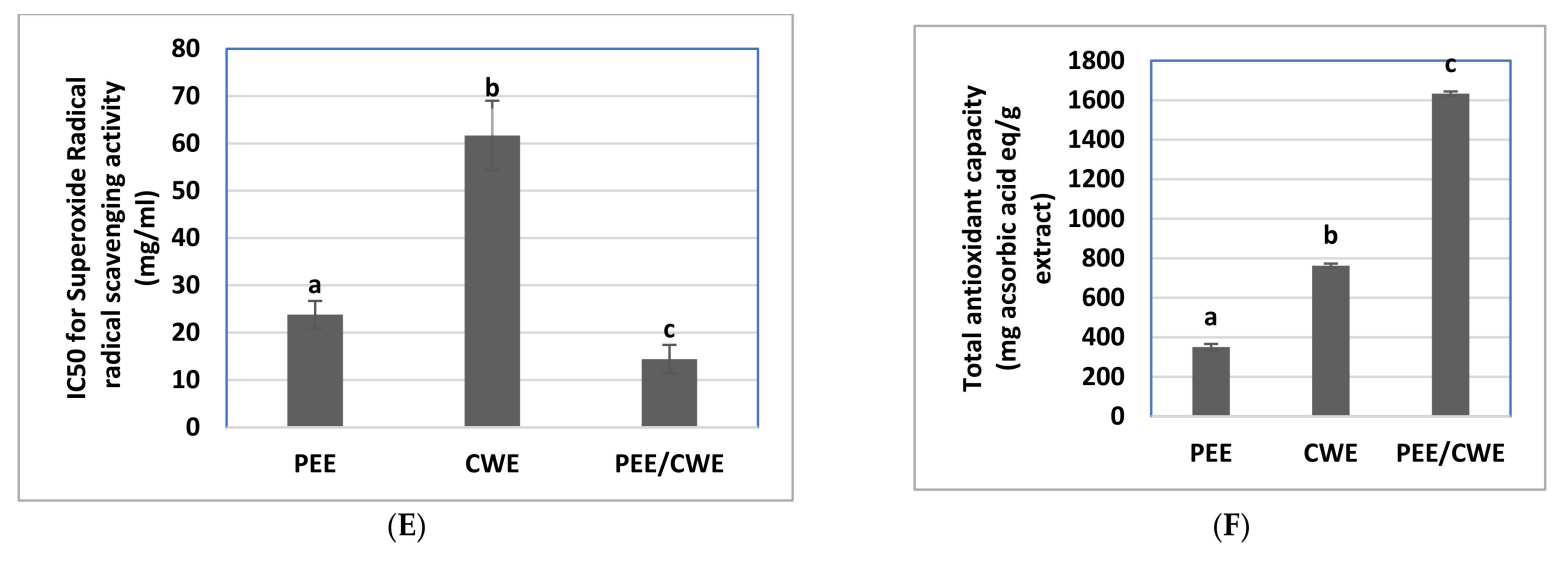
HPLC analysis and in vitro antioxidant activities of Purslan ethanolic extract (PEE), Chicory water extract (CWE), and their combination (PEE/CWE). (**A**) HPLC analysis of PEE. (**B**) HPLC analysis of CWE. (**C**) α,α-diphenyl-β picrylhydrazyl (DPPH) scavenging activity. (**D**) Nitric oxide (NO) scavenging activity. (**E**) Superoxide radical scavenging activity. (**F**) Total antioxidant capacity. The results are shown as the mean ± SD (*n* = 3). Different letters for the same parameter are significantly different at *p* < 0.05.

The current results demonstrated that PEE and CWE contain various phytochemical constituents, minerals, and vitamins with different concentrations, as shown in Table 2. PEE contains a higher content of total phenolics and flavonoids and represents a higher yield than CWE. Additionally, the HPLC analysis of PEE revealed the presence of many phenolic and flavonoid compounds, where benzoic acid, p-hydroxybenzoic acid, myricetin, quercetin, rosmarinic acid, catechol, syringic acid, chlorogenic acid, p-coumaric acid, and gallic acid exist in great amounts. At the same time, only resveratrol and naringin are present in CWE in a large amount (Figure 2A,B and Table 2).

Moreover, the minerals and vitamin analyses indicate that PEE and CWE are mineral- and vitamin-rich nutrients due to the presence of Na, K, Ca, Fe, Mg, and P in great amounts, with Cu, Mn, Zn, and Se in the least amount. PEE contains a greater amount of vitamin A, while CWE includes a higher amount of folic acid and vitamin C, Table 2.

### 3.2. In Vitro Antioxidant Properties of PEE, CWE and Their Combination

The radical scavenging ability of PEE and CWE to DPPH, NO, and superoxide radicals, as well as the total antioxidant capacity, are illustrated in (Figure 2C–F). PEE has a potent scavenging ability that was significantly (*p* *˂* 0.05) superior (lower IC50 value) than that of CWE, associated with a significant (*p* *˂* 0.05) higher total antioxidant capacity than that of CWE. Concerning the synergistic analysis, the combination of PEE and CWE (PEE/CWE) showed a synergistic (CI ˂ 1) scavenging effect against DPPH, NO, and superoxide radicals (Table 3). Moreover, the total antioxidant capacity was synergistically enhanced upon combining these extracts.

### 3.3. The Effects of Different Treatments on Semen Quality, Fructose Level, α-Mannosidase Activity, and Hormonal Levels

The results of the effects of supplementation treatments on semen quality are presented in Table 4. Exposure to long-term Dexa injections for constitutive six weeks has a significant impact on sperm quality (*p* < 0.05). Although the Dexa-injected group showed a significant increase in the sperm count, this increase was associated with a significant decrease in the morphology index, sperm progressive motility, and % motility during the 1st, 2nd, and 3rd hours, with a significant increase in the non-progressive motility relative to values of the control group (*p* < 0.05). The groups injected with Dexa and supplemented with PEE, CWE, or their combination had a significant (*p* < 0.05) improved semen quality as compared to the Dexa-untreated group (Table 4). The synergy testing (Table 3) revealed a synergistic (CI < 1) effect of PEE/CWE for all semen quality biomarkers, except for sperm count, for which it showed an additive effect (CI = 1).

Additionally, the negative effect of Dexa on sperm morphology index and motility could probably be due to the observed reduced fructose levels and α-mannosidase activity. Dexa injections for 6 weeks showed a significant (*p* < 0.05) decline in serum and seminal fructose levels and seminal α-mannosidase activity relative to the control group. In comparison, the three treated groups (Dexa + PEE, Dexa + CWE, and Dexa+ PEE/CWE) illustrated a significant (*p* < 0.05) elevation in these markers relative to the Dexa group. Concerning the hormonal status, the Dexa-injected untreated group revealed a significant (*p* < 0.05) decrease in serum levels of male hormones (FSH, LH, and testosterone) relative to the control group. On the other hand, administration of either PEE, CWE, or their combination to Dexa-injected rats resulted in a significant (*p* < 0.05) increase in FSH, LH, and testosterone levels relative to the Dexa group. The combination of PEE and CWE (Dexa+ PEE/CWE group) synergistically (CI < 1) elevated the α-mannosidase activity, FSH, LH, and testosterone levels (Table 4) and additively affected (CI = 1) the fructose level (Table 3).

### 3.4. The Effects of Different Treatments on Testicular Oxidative Stress Markers

As shown in Table 4, a marked elevation in testicular MDA level accompanied by significant decreases in GSH content and GPx and GST activities were noticed in the Dexa-untreated group relative to the control group (*p* < 0.05). In contrast, a marked decrease in testicular MDA level and concurrent increases in GSH content and GPx and GST activities were observed in all treated rats. The combination of PEE and CWE revealed a synergistic (CI < 1) antioxidant effect, as indicated for all the studied oxidative stress parameters (Table 3). These results suggest a better aptitude for the combined treatment (PEE/CWE) to fight oxidative stress in the testicular tissues. In the present study, the positive effects of the PEE/CWE combination on semen quality and spermatogenesis can be attributed to the potential antioxidant activity of these extracts.

### 3.5. The Effects of Different Treatments on Glycemic Markers

**Figure 3:**
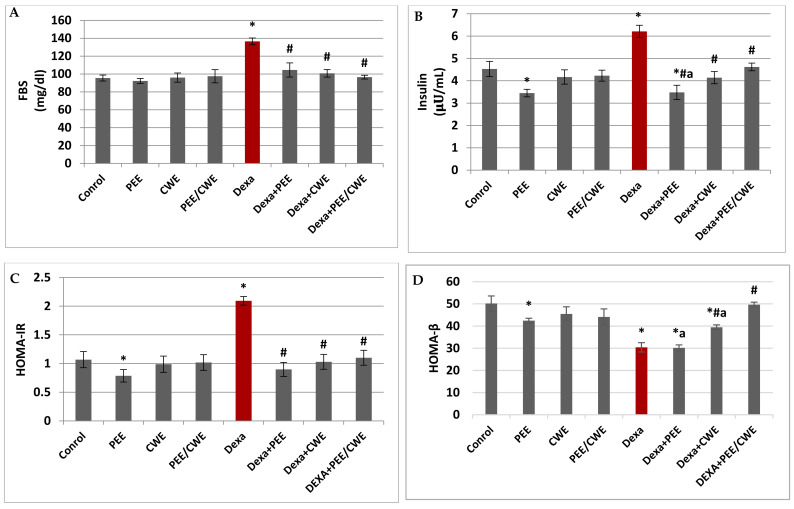
The effects of different treatments on serum glycemic markers. (**A**) Fasting blood glucose (FBG). (**B**) Insulin level. (**C**) HOMA-IR. (**D**) HOMA-β. Data are expressed as the mean ± SD of six rats. Two-way analysis of variance (ANOVA) was used, followed by Tukey’s post hoc test (* *p* < 0.05 vs. control group, ^#^ *p* < 0.05 vs. Dexa group, and ^a^ *p* < 0.05 vs. DEXA + PEE + CWE group).

The FBG and insulin levels in the Dexa-injected group displayed a significant increase accompanied by a significant elevation in HOMA-IR level and a significant reduction in HOMA-β level versus the control group (*p* < 0.05; Figure 3). In contrast, the administration of PEE, CWE, or their combination for eight constitutive weeks to Dexa-injected rats significantly (*p* < 0.05) reduced the FBG, insulin, and HOMA-IR levels relative to the Dexa-untreated group. Furthermore, the CWE and PEE/CWE treated groups showed a significant increase in the HOMA-β index compared to both the Dexa and PEE + Dexa groups. Moreover, the Dexa + PEE/CWE group reached levels comparable to the control group (Figure 3). Interestingly, the combination of PEE and CWE showed a synergistic (CI < 1) effect for all the studied glycemic markers, except for the HOMA-β index, which showed an antagonistic effect (CI > 1; Table 3). Therefore, long-term Dexa administration may indirectly promote male infertility via hyperglycemia. In contrast, PEE/CWE may improve male fertility via effects on circulating glucose and insulin levels.

### 3.6. The Effects of Different Treatments on ER Stress Markers and Autophagy Regulators

**Figure 4:**
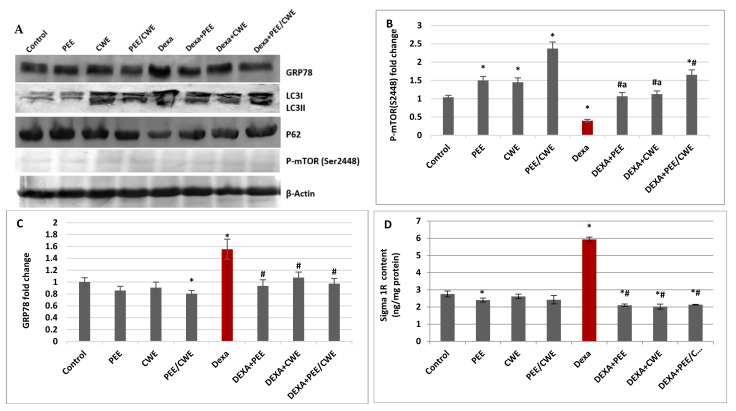

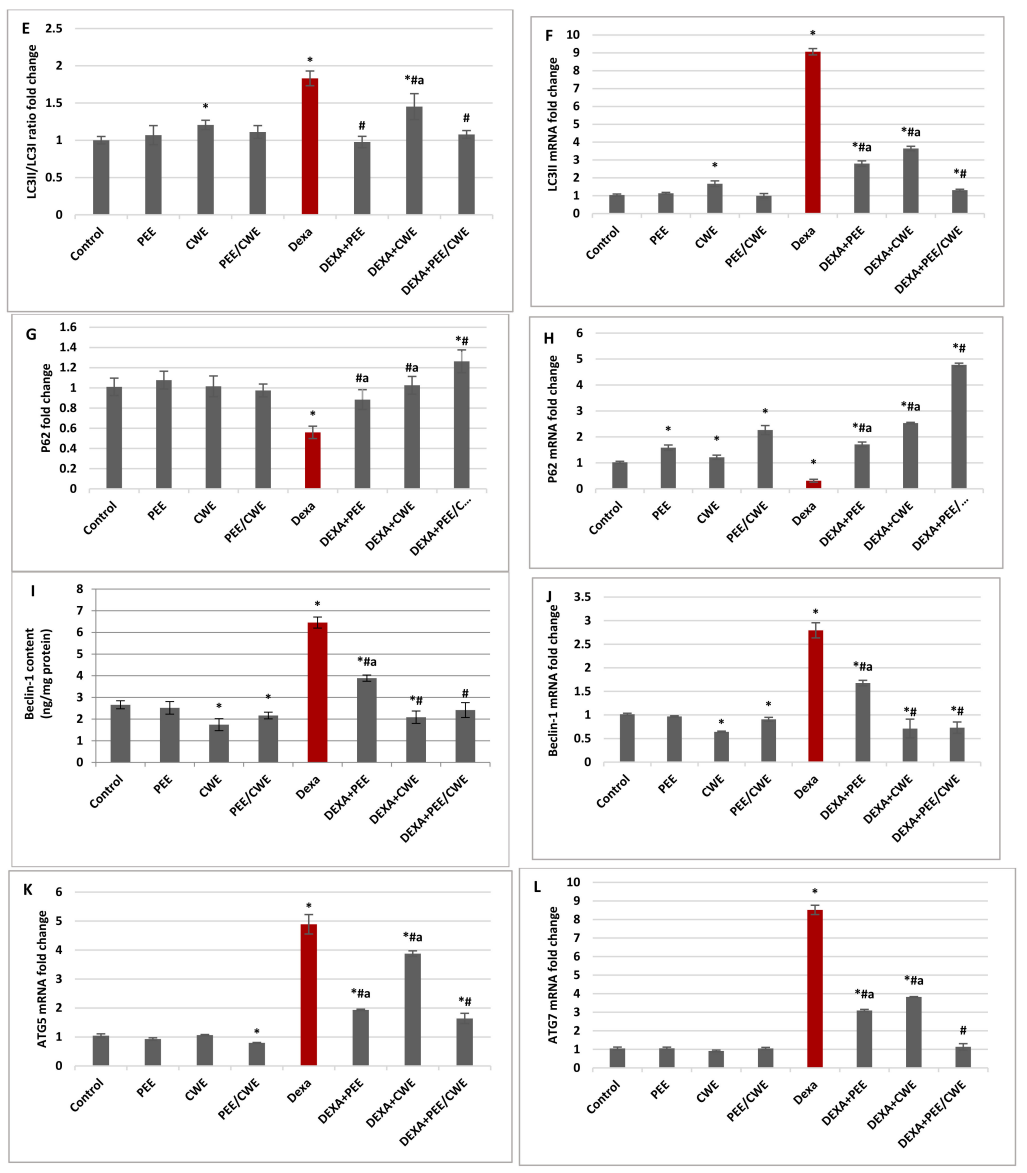

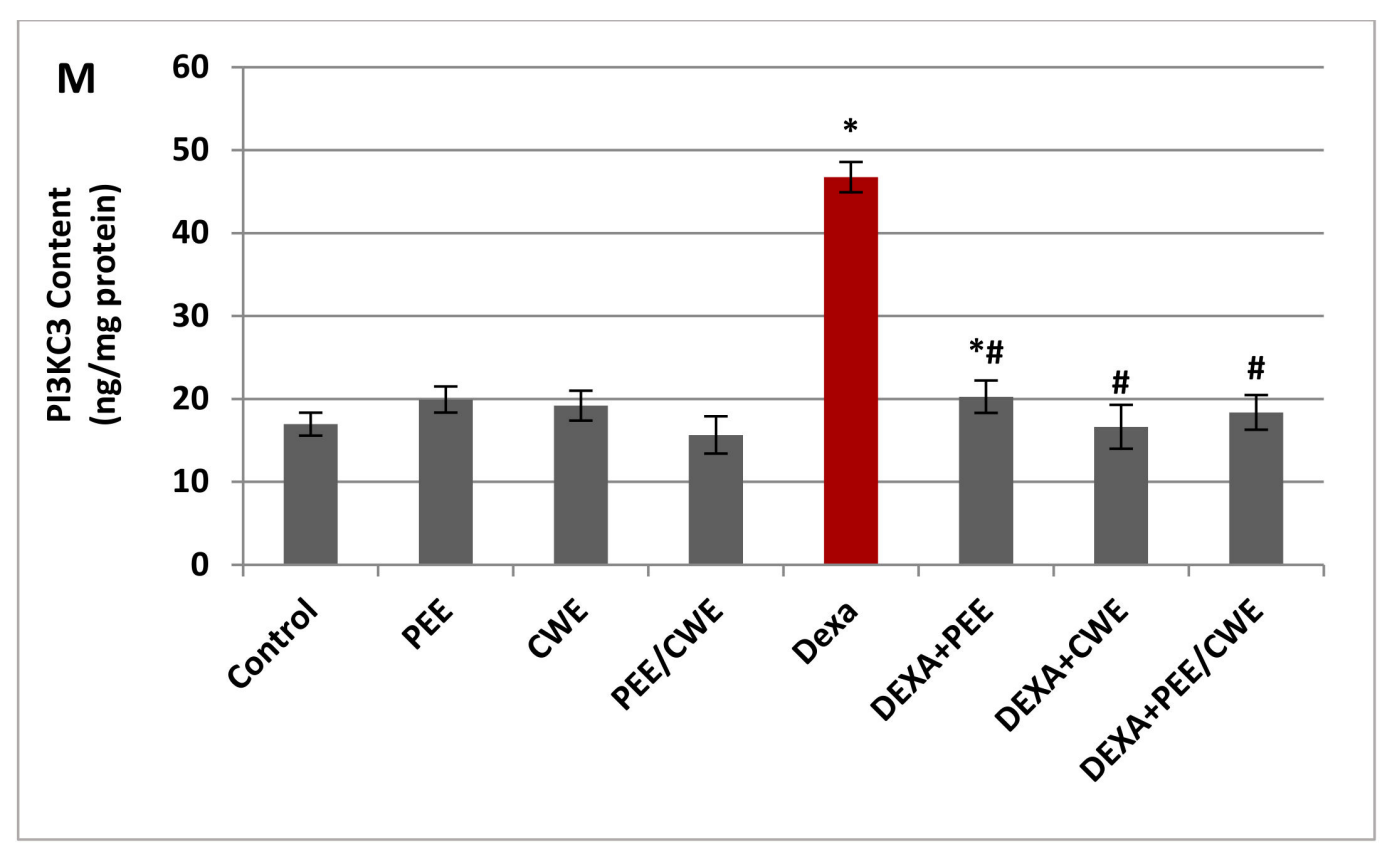
The effects of different treatments on RE stress and autophagy markers. (**A**) Immunoblots for changes in protein expression of GRP78, LC3, P62, and P-mTOR(S2448), while β-actin served as an internal control. (**B**) P-mTOR(S2448) protein fold change. (**C**) GRP78 protein fold change. (**D**) Sigma 1R protein content. (**E**) LC3II/LC3I ratio protein fold change. (**F**) LC3II mRNA fold change. (**G**) P62 protein fold change. (**H**) P62 mRNA fold change. (**I**) Beclin-1 protein content. (**J**) Beclin-1 mRNA fold change. (**K**) ATG5 mRNA fold change. (**L**) ATG7 mRNA fold change. (**M**) PI3KC3 protein content. Data are expressed as the mean ± SD of six rats. Two-way analysis of variance (ANOVA) was used, followed by Tukey’s post hoc test (* *p* < 0.05 vs. control group, ^#^ *p* < 0.05 vs. Dexa group, and ^a^ *p* < 0.05 vs. DEXA + PEE + CWE group).

Sigma 1R and GRP78 are important contributors to the ER stress response. A significant (*p* < 0.05) elevation in the protein levels of GRP78 and Sigma 1R was detected in the testicular tissues of Dexa-induced infertile rats relative to the control group (Figure 4C,D). On the other hand, treatment of Dexa-injected rats with either PEE, CWE, or PEE/CWE significantly (*p* < 0.05) attenuated the increased GRP78 and Sigma 1R protein levels relative to the Dexa-untreated group.

To investigate the involvement of Dexa and ER stress in autophagy modulation, the autophagosomal regulators, including PI3KC3, ATG5, ATG7, Beclin-1, LC3-II, P-mTOR(S2448), and P62, were detected at the protein and/or gene expression levels. Concerning the autophagy initiation and processing, the PI3KC3 protein level (Figure 4M) and autophagy-related genes, including ATG5 and ATG7 mRNA expression levels (Figure 4K,L), were significantly (*p* < 0.05) elevated in Dexa-injected rats compared to the control rats. LC3II (Figure 4E,F) and Beclin-1 (Figure 3I,J) mRNA and protein expression levels were significantly (*p* < 0.05) elevated in the Dexa-induced group compared to the control group. On the other hand, the quantitative RT-PCR analysis indicated a significant (*p* < 0.05) downregulation of the p62 mRNA expression level (Figure 4H) in Dexa-injected rats, and the densitometric analysis of the Western blot (Figure 4G) confirmed the significant (*p* < 0.05) reduction in P62 protein level compared to the control group. The P-mTOR(S2448) protein level (Figure 4B) was significantly (*p* < 0.05) reduced in the Dexa-injected rats compared to the control rats. Together, these results indicated that the autophagy rates are enhanced in the Dexa-injected rats and that autophagy may be involved in the pathogenesis of male infertility.

The expression of autophagy-related proteins following PEE, CWE, or PEE/CWE treatment was evaluated to delineate their role in alleviating Dexa-induced testicular toxicology. Administration of PEE and CWE to the Dexa-injected rats attenuated the autophagy and introduced a significant (*p* < 0.05) ameliorating effect in these parameters compared to the Dexa-untreated group. The combined impact of the PEE/CWE combination on reducing the autophagy process was greater than the individual treatments with either PEE or CWE. P-mTOR(S2448), P62, Beclin-1, LC3-II, ATG5, and ATG7 revealed a synergistic effect (CI < 1), while PI3KC3 showed an additive effect (CI = 1). These outcomes suggest that combinations of PEE with CWE may synergize the inhibition of Dexa-induced male infertility through attenuation of ER stress and autophagy.

### 3.7. Histological Changes and Morphometric Analysis in Testicular Tissues of Different Experimental Groups

**Figure 5:**
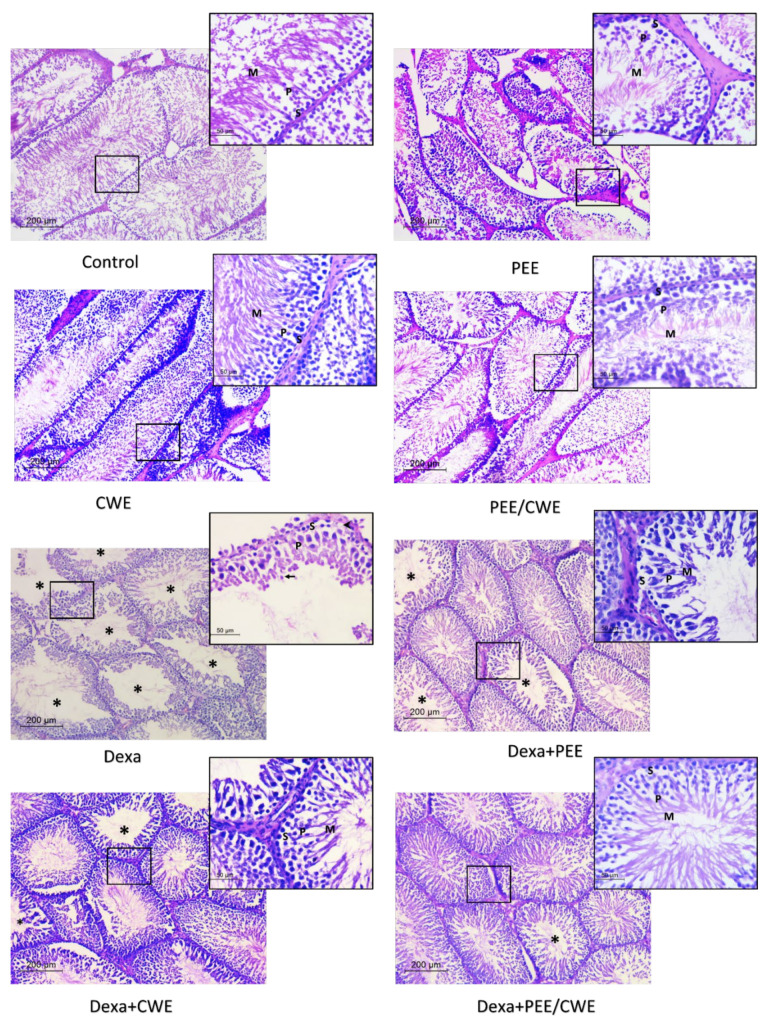
H&E-stained histologic sections of seminiferous tubules in testicular tissues of the different studied groups. Control, PEE, CWE, or their combination showed mature sperms in all tubules (high spermatogenesis index). Higher magnification showed normal spermatogonia (S), multiple layers of spermatocytes (P), and mature sperms (M). The Dexa-treated model showed tubules with wide lumens lacking mature sperms (*). Higher magnification showed degenerated spermatogonia with focal cytoplasmic vacuolation (arrowhead), few spermatocytes (P), spermatids (arrow), and no sperms. Dexa + PEE and Dexa + CWE showed an improved spermatogenesis index and tubular lining in high magnification. The Dexa-treated group received combined products, and most tubules showed active spermatogenesis and intraluminal sperms. No degenerative changes were detected under high magnification. *: tubules with wide lumens and absent sperms; S: spermatogonia; P: spermatocytes; M: mature sperms; arrowhead: degenerative changes; arrow: spermatids. H&E; ×00, insert ×400).

Figure 5 illustrated that control group rats showed normal testicular tissue. Most of the tubules showed active spermatogenesis (the spermatogenesis index ranged between 95 and 98%). They were lined by thick germinal epithelium composed of viable spermatogonia followed by multiple layers of mitotically active spermatocytes. Lumens showed mature sperms with their tails projecting to the lumen (Figure 5). Other control groups that received PEE, CWE, or their combination revealed normal histology and an SI between 94 and 98%.

The Dexa-treated group showed major changes in testicular histology. Seminiferous tubules showed wide lumens, which were lined by disorganized thinned epithelium. Spermatogonia showed degenerative changes in the form of cytoplasmic vacuolation and nuclear hyperchromasia (Figure 5). Maturation arrest at the level of spermatocytes or spermatids was seen in most tubules with no sperms in the lumens. Vascular congestion was also noted in the stroma. The SI ranged between 50 and 61% in the different examined rats.

The PEE + DEXA and CWE + DEXA groups showed a protective effect on seminiferous tubules. The SI increased to be 70–73% and 75–78%, respectively. Improvement in testicular morphology was also seen in the increased thickness of the epithelial lining. No degenerative changes were detected. Moreover, the combined treatment of both products (Dexa + CWE/PEE) showed a better protective effect on Dexa-treated group (Figure 5). The SI index increased to 80–85%, with the restoration of active spermatogenesis in most seminiferous tubules.

## 4. Discussion

Synthetic GCs are important long-acting steroid hormones used to treat everything from severe infection, asthma, to arthritis. Most patients are treated with low doses over a long period. However, long-term or high-dose usage of GCs can lead to a series of side effects [75]. Dexa at doses as low as 250 μg daily can decrease testicular adrenal rest tumor size and reverse male infertility [76]. At the same time, chronic administration of Dexa was reported to produce large amounts of reactive species and is suggested to induce oxidative stress [8]. Oxidative stress and excessive ROS accumulation are considered major causative factors of insulin resistance, ER stress, and autophagy dysregulation, causing testicular degeneration and dysfunction [22,23]. Therefore, GCs are a double-edged sword for treatment [75]. The current study explored the synergetic protective effect of combining two edible Egyptian plants (Purslane and Chicory) against Dexa-induced testicular damages. Purslane and Chicory extracts are rich sources of phenolics, flavonoids, minerals, and vitamins, with potent antioxidant properties.

Several studies have revealed that Dexa acts as a testicular toxicant. Dexa induces histopathological alterations and spermatogenesis defects, including epithelial vacuolizations, reduction in seminiferous tubule diameter, the elevation of the apoptotic index of germ cells, poor spermatogenesis, and significant maturation arrest [7,59]. Prolonged exposure to exogenous GCs has been linked to a rapid decline in testosterone levels [7,77,78]. Normally, the brain’s hypothalamus produces gonadotropin-releasing hormone (GnRH), which stimulates the pituitary gland to produce LH and FSH. LH stimulates testosterone production by binding to its receptor on Leydig cells in the testis. FSH binds to its receptor in the Sertoli Cell within the seminiferous tubule to drive spermatogenesis and alter the quantity of LH receptors through paracrine control [79]. Testosterone is essential for the onset of male puberty and spermatogenesis and the maintenance of secondary sexual characteristics [80]. Moreover, testosterone and FSH enhance the production of regulatory molecules and nutrients necessary to maintain spermatogenesis. As a result, testosterone and FSH are required for full reproductive potential [81]. In contrast, an elevated level of GCs inhibits the testicular steroidogenesis directly by affecting the differentiation of the Leydig cells [80] and indirectly via stimulating the expression of the gonadotropin-inhibitory hormone, which negatively regulates the HPG axis and reduces GnRH production, as well as directly lower pituitary LH and FSH secretion, which suppresses testosterone production [82]. Mohammed, Mansour [78] reported that oral Dexa at a lower total cumulative dose of 240 mg resulted in hypogonadism in male patients.

Accordingly, in the current study, Dexa injections for six constitutive weeks impaired spermatogenesis function and negatively affected the sperm morphology index and motility with a non-significant effect on the sperm count compared to the control group. Several studies indicated that the Dexa treatment decreases sperm motility [7,59] due to the interruption in ATP production resulting from oxidative stress, which affects sperm motility and causes the death of sperms [59]. Furthermore, the Dexa group revealed a marked decline in the levels of serum testosterone, LH, and FSH relative to the control group, which is in accordance with previous studies [82,83]. Moreover, Dexa caused dramatic alterations in the testicular tissues’ histological structure, consistent with earlier animal studies [10,28,84]. These disturbances could be attributed to the lack of gonadotropin, severe oxidative stress, and Leydig cell loss [28,85].

The detrimental effect of Dexa on the spermatogenesis index may also be due to the reduced fructose level and α-mannosidase activity. Fructose is the main sugar related to metabolism and sperm motility. It is an important energy source and a marker of the performance of seminal vesicles. Testosterone regulates seminal fructose levels by controlling the secretion function of the accessory glands [17,86]. As a result, a depleted testosterone level causes a drop in semen fructose, affecting normal sperm motility. Moreover, α-mannosidase is one of the lysosomal glycosidases in mammals’ male genital tract and semen. Mannosidases are associated mainly with reproductive and Sertoli cells and play a significant role in spermatozoa maturation [87]. Moreover, α-mannosidase is implicated as a non-catalytic binding protein during sperm-oocyte interaction. Hence, depleted α-mannosidase activity in spermatozoa may adversely affect spermatozoa maturation and fertilization [88].

In the male reproductive system, ROS exhibits a dual role. At minimal physiological concentrations, ROS function as signaling molecules and exerts a vital physiological role in regulating the HPG axis, steroidogenesis, and spermatogenesis, as well as enhancing sperm capacitation, hyperactivation, and acrosome reactions. However, overproduction of ROS pushes the cellular redox balance towards an oxidative state linked to several male fertility problems, including varicocele, leukocytospermia, and idiopathic infertility [18,89]. Therefore, oxidative stress can be one possible contributor to the etiology of male infertility [17]. Oxidative stress-related mechanisms are assumed responsible for altering male reproductive indices in about 40% of cases [19,27]. In addition to the excessive ROS formation, the scarcity of cytoplasmic antioxidants is the primary cause of oxidative stress [90]. Spermatozoa are vulnerable to ROS attack as they contain a considerable amount of polyunsaturated fatty acids and are rich in mitochondria. Therefore, Spermatozoa are more susceptible to oxidative stress, which may initiate lipid peroxidation, promote peroxidative damage, result in sperm motility loss, disrupt the androgen-producing Leydig cells, and increase oxidative DNA damage [84,90,91]. 

Previous studies showed that Dexa induces excessive ROS production and testicular oxidative stress in rodents [28,84]. Lipid peroxidation is one of the predominant processes resulting from oxidative stress. MDA is a well-known secondary lipid peroxidation product that can be utilized to detect cell membrane damage [17]. In contrast, the body’s antioxidant defense capacity (GSH, GPx, and GST) scavenges the free radicals. GSH is vital in the fight against lipid peroxidation. GSH acts as a cofactor for GPx and serves as a reductant and a scavenger nucleophile [92]. Moreover, GPx is the second line of protection; it inactivates hydroperoxides (H_2_O_2_ and lipid peroxides) at the expense of GSH. Furthermore, GSTs are important phase II detoxification enzymes that catalyze the conjugation of GSH with various electrophilic compounds [93]. In the present study, a significant decrease in the testicular GSH level and GPx and GST activities was observed in the Dexa-treated animals, which are in accordance with Hasona [28], Mukherjee, Haldar [84]. GSH depletion led to the inhibition of GPx and GST activities. Furthermore, this decline could contribute to the stimulation of lipid peroxidation, which was confirmed by a significant increase in the testicular MDA level.

Increased oxidative stress appears to be a probable signal for insulin resistance, which can be blocked by a wide range of antioxidants [94]. In the current study, the significant decrease in antioxidant levels and the increased lipid peroxidation in the Dexa-treated rats showed a positive correlation with the increased insulin resistance, as evident from the significant increase in FBG, insulin, and HOMA-IR levels, also accompanied by a significant reduction in the HOMA-β level. Dexa-induced insulin resistance was investigated in several tissues by different mechanisms, including the inhibition of glucose uptake and the reduction of glucose oxidation with increasing proteolysis, lipolysis, steatosis, gluconeogenesis, and hyperglycemia [95,96,97,98]. Studies have demonstrated that GC administration at high doses and/or chronic periods (days to weeks) promotes dysregulation of hepatic glucose metabolism, which is directly related to the reduction of insulin action in the liver, which ultimately means hepatic GC-induced insulin resistance [99,100].

In addition, the present study showed that autophagy was over-activated in the testis of Dexa-administered rats, implying that autophagy may play a role in Dexa-induced spermatogenesis deficiency. Autophagy, or programmed cell death type II, is an evolutionarily conserved process that involves the construction of a double-membrane vesicle called an autophagosome, fusion with lysosomes, and destruction of cytosolic components by resident hydrolases [101,102,103]. Increasing evidence illustrates that autophagy was activated in human spermatozoa and involved a broad range of cellular events within the male reproductive system to regulate cell survival and motility [104]. Low-level autophagy under normal physiological conditions is crucial for forming specific structures that guarantee successful spermatogenesis and the degradation of certain constituents [24]. Cumulative studies reveal that autophagy is a double-edged sword, where excessive autophagy, which can be activated by various extracellular or intracellular stimuli, can destroy the basic components of cells and lead to “autophagic cell death” [25,105]. Autophagy is triggered by severe oxidative stress, and it aggravates the testicular oxidative damage [23,105]. Therefore, we explore the positive significance of the effect of Dexa on the oxidative stress–autophagy axis in the male reproductive system. It has been reported that autophagy can be abnormally activated by increased ER stress and eventually lead to cell death [106]. GRP78 is a master regulator of ER stress and is required for stress-induced autophagy. GRP78 plays a critical role in maintaining cell life in case of stress [107]. Sigma 1R is an important gatekeeper that keeps ER stress under control. Sigma 1R is activated under ER and oxidative stresses to activate antistress and antioxidant response elements and attenuate the formation of ROS [108]. Normally, Sigma1R is complexed with GRP78 on the ER membrane. ER stress induces the dissociation of Sigma1R from GRP78 and promotes their translocation to the whole ER network to inhibit ER stress-induced apoptosis [109]. Our study showed that the expression levels GRP78 and Sigma 1R were significantly increased in the Dexa-untreated group, suggesting that Dexa can induce ER stress, consistent with Duzgun, Bedir [107] and Zode, Sharma [110] in different experimental models.

Concerning the autophagic process, ATG5, ATG7, and LC3 are involved in phagophore expansion and promote the formation of autophagosomes. ATG5 and ATG7 are two important autophagy-related proteins as they participate in the initiation of autophagosome formation. Overexpression of ATG5 or ATG7 can increase autophagic activity as a pro-survival mechanism to protect cells under stress conditions [111]. LC3 is one of the most widely used markers of autophagy. LC3 is hydrolyzed to form LC3I, which is further activated by ATG7 and modified by phosphatidylethanolamine to form LC3II. LC3II production is a classical hallmark of autophagy that involves fusing autophagosomes with the lysosome to create autolysosomes [112]. Therefore, the ratio of LC3II/LC3I and the accumulation of the LC3II results in an increase in autophagosomes and is often used to evaluate autophagy [111]. The PI3KC3/Beclin-1 complex is critical in the early stage of autophagosome formation to nucleate the phagophore [113]. Beclin-1 is important for the localization of autophagic proteins to a pre-autophagosomal structure through interaction with PI3KC3/Vps34, forming the Beclin-1–Vps34–Vps15 core complex, which is the nucleation complex [114,115]. Another key regulator of autophagy is the mammalian target of rapamycin (mTOR), an autophagy inhibitor. mTORC1 contains mTOR phosphorylated predominantly on S2448. A reduced P-mTOR(S2448) level activates Beclin-1 and promotes LC3I lipidation to produce LC3II that localizes to the autophagosome membrane, enabling the autophagophore elongation and forming autophagosomes [116]. P62 is an essential autophagy adaptor, also known as P62/SQSTM1. P62 is served as a selective autophagy receptor involved in the removal of ubiquitinated proteins and the formation of the autophagosome. The P62 protein level is usually negatively correlated with autophagic degradation. When the autophagic flux is blocked, many ubiquitinated proteins accumulate in the cells, and the level of P62 protein will increase [117,118].

Herein, Dexa upregulated the autophagy-associated genes, including ATG5, ATG7, PI3KC3, Beclin-1, LC3II, and the LC3II/LC3I ratio, associated with downregulation of the P-mTOR(S2448) and P62 protein levels. Together, these results indicate that the autophagy rates are enhanced in Dexa-injected rats, and that autophagy hyperactivation may be involved in the pathogenesis of male infertility. Therefore, Dexa demonstrated a rat model of testicular damage through activation of testicular oxidative stress and insulin resistance that aggravate ER stress and autophagic degradation.

A diet rich in antioxidants significantly reduces the incidence of several oxidative stress-related diseases. Plant phenolics are vital human dietary secondary metabolites that exhibit antioxidant properties and enhance human health. Plant phenolics are effortlessly absorbed in the intestine with relatively low toxicity and cost [119,120]. Phenolic acids, a subclass of plant phenolics, possess phenolic hydroxyl groups as the antioxidant core. The number and position of phenolic hydroxyls lead to the variation in their antioxidant potential. Therefore, different phenolic acids have various antioxidant activities [121]. The phenol moiety and resonance stabilized the structure in phenolic acids, resulting in radical scavenging via H-atom donation, radical quenching via electron donation, and singlet oxygen quenching [119]. Therefore, phenolic acids are thought to be powerful antioxidants that protect the body from free radical damage and chronic illnesses [121]. PEE and CWE are rich sources of different polyphenolic compounds [37,122]. The current study illustrated that PEE contains plenty of phenolic acids, including benzoic acid or hydroxybenzoic acid derivatives (p-hydroxybenzoic acid, rosmarinic acid, syringic acid, and gallic acid) and cinnamic acid derivatives (chlorogenic acid and p-coumaric acid) in addition to flavonoids such as myricetin, quercetin, and catechol. At the same time, CWE has a large amount of polyphenol resveratrol and flavonoid naringin. Moreover, PEE and CWE are vitamin- and mineral-rich nutrients. Vitamin A and C, as potent antioxidant vitamins, are present in adequate amounts in PEE and CWE, respectively, in addition to the presence of folic acid in CWE, which is important in DNA production and cell division. Therefore, the PEE and CWE combination (PEE/CWE) has a synergistic (CI ˂ 1) radical-scavenging ability against DPPH, NO, and superoxide radicals, and thus have total antioxidant capacity. These results agree with the previous studies that have shown the antioxidant properties of PEE and CWE [37,122,123,124].

Furthermore, essential trace minerals in PEE and CWE empower their antioxidant potential. K, Ca, Fe, Mg, Cu, Mn, Zn, and Se participate in proper protein folding and stability and activate the common antioxidant enzymes, including Cu/Zn/Mn-Fe SOD and Se-GPx, as well as catalases and peroxidases that require Fe and Mg [125,126,127]. Interestingly, α-mannosidase, which is essential in spermatozoa maturation, is a Zn-dependent enzyme and the active Zn–protein complex is readily dissociable at the pH of optimum enzyme activity (pH 5) [128]. Moreover, in the context of male reproduction, Zn is a potent antioxidant, and its lack in diets is known to cause oxidative stress in spermatozoa and reduce male fertility [90]. Collectively, the antioxidant compounds, vitamins, and minerals play a key role in peroxide detoxification, combating oxidative stress, preventing cell membrane damage, and enhancing α-mannosidase activity. Therefore, balanced oxidative stress parameters (MDA and GSH levels and GPx and GST activities) were re-established in the testicles after administration of PEE and CWE, resulting from all these factors working together. Harmonious with these findings, previous studies illustrated the antioxidant and health-improving activities of Purslane [122,123,124,129] and Chicory extracts [130,131,132]. Thus, administration of PEE and CWE along with Dexa-enhanced antioxidative enzyme activities, suppressing lipid peroxidation and thus rescuing testes from Dexa-induced oxidative load.

Moreover, the current study revealed that long-term Dexa administration might indirectly promote male infertility via hyperglycemia. In contrast, administering PEE and CWE ameliorates male infertility by improving glycemic markers. Our results provide strong support for previous studies suggesting the antidiabetic actions of Purslane [124,133] and Chicory extracts [134,135]. Interestingly, Chicory root is the most abundant source of dietary fiber inulin, representing about 68% of the total compounds in fresh Chicory roots. Inulin is a polymer of fructose with a β-(2-1)-glycosidic bond, is low in calories, and can be used as a sugar substitute [37]. It is also an excellent component of a diabetic diet and was reported to improve glucose and lipid metabolism [136,137,138]. Moreover, Rahimiyan-Heravan, Roshangar [138] reported the potential therapeutic effects of inulin on serum and testicular reproductive markers in diabetic male rats.

In the current study, administration of PEE and CWE reduced Dexa-induced testicular abnormalities and histological changes in rats. PEE and CWE successfully ameliorated the HPG axis dysfunction by restoring the serum testosterone, LH, and FSH levels. A remarkable improvement in the spermatogenesis markers, including sperm count, motility and morphology index, fructose levels, and mannosidase activity, also was noticed compared to the Dexa-untreated group. This ameliorative effect of PEE and CWE might be due to the presence of flavonoid constituents, minerals, and vitamins that improve the antioxidant levels and have a positive effect on the male reproductive activity. In addition, the CWE-administered groups showed improved fructose levels, androgenicity, and glycemic markers compared to PEE, which may be due to the presence of inulin. This finding was in line with the finding of Dorostghoal, Seyyednejad [39], where Chicory leave extract was reported to improve the reproductive parameters in male rats due to its antioxidant and androgenic properties. No earlier studies deduced that PEE enhanced male reproductive potential. Ahangarpour, Lamoochi [139] demonstrated the attenuation of female sex hormones in aging mice after administration of PEE due to its antioxidant potential. While Nayaka, Londonkar [140] demonstrated the anti-fertility effect of Purslane chloroform extract in female albino rats. Okafor, Nnamah [141] also reported the reproductive toxicity potentials (spermatotoxicity and sperm maturation arrest potential) of Purslane stem and leaf methanolic extracts in male rats, as indicated by reduced sperm count and motility at higher doses (400 and 800 mg/kg).

Moreover, the effect of PEE and CWE on ER stress and autophagy has not been studied yet. The current study finds that PEE and CWE suppressed ER stress and autophagosome formation and significantly attenuated Dexa-induced testicular toxicity. The current treatments introduce a significant decline in GRP78 and Sigma 1R levels. These findings suggest that pretreatment with PEE and CWE can act as ER stress inhibitors, significantly suppress Dexa-induced autophagy, and reduce the induced testicular damage. Furthermore, the results demonstrated that the expression levels of P-mTOR(S2448) and P62 were enhanced by PEE and CWE, leading to decreased expression of ATG5, ATG7, PI3KC3, Beclin-1, and LC3II/LC3I. Previous studies are consistent with the current findings where the inhibition of ER stress can significantly inhibit autophagy’s overactivation and ultimately reduce testicular damage [23,104,107]. Recently, Purslane water extract alleviated inflammatory bowel disease by regulating ER stress and autophagy [142].

PEE and CWE are multitarget agents whose pharmacological response modulates numerous targets/pathways, which may be beneficial against male infertility. Therefore, we evaluated the protective effect of the combination between PEE and CWE against Dexa-induced testicular toxicity. CI values indicated that this combination exhibited synergistic action (CI ˂ 1) in most studied parameters. As previously published, PEE is an effective antioxidant and hypoglycemic mediator [133]. In comparison, CWE is safe and effective in improving testicular oxidative status, androgenicity, and spermatogenesis [39]. Interestingly, the efficacy of either PEE or CWE is enhanced by their combination may be due to the summation of their constituents, including phenols, flavonoids, polysaccharides, minerals, and vitamins. Therefore, PEE and CWE can be combined with GCs therapy to protect male reproductive potential. Collectively the current data show the health-promoting properties of these widely consumed salad plants.

## 5. Conclusions

Dexa was found to negatively regulate the HPG axis and suppress the secretion of LH and FSH, which reduced testosterone production, consequently leading to depletion of the fructose level, and finally impairing the spermatogenesis function and the normal sperm motility. Interestingly, the current findings provide novel insight into the mechanism of Dexa-induced testicular dysfunction in male rats. Besides the androgen deficiency, oxidative stress, ER stress, and autophagy hyperactivation play a crucial role in Dexa-induced testicular toxicity. Inhibiting oxidative stress and ER stress (reduced GRP87 and Sigma 1R) using PEE and CWE remarkably blocked the Dexa-induced autophagy (elevated the P-mTOR(S2448) and P62 levels and reduced the ATG5, ATG7, PI3KC3, Beclin-1, and LC3II/LC3I expression levels). A combination of PEE/CWE proposed a synergistic (CI < 1) antioxidant and ameliorating capacity. Therefore, from a clinical perspective, combinatorial therapy of PEE/CWE along with GCs may show improved results for managing male reproductive health and delay the progression of testicular toxicity. As the mechanism of Dexa-induced autophagy is not fully elucidated, the present communication proposes enhanced oxidative stress to be an important factor, besides hormonal imbalance, in mediating Dexa-induced testicular toxicity. However, deeper insights into the autophagic and apoptotic pathways operating therein are required for a broader elucidation of the mechanism(s) by which oxidative stress augments the Dexa-induced testicular toxicity.

## Figures and Tables

**Table 1:** Primers’ sequence and qRT-PCR conditions.

Gene Name/ Accession Number	Primer Sequence		Annealing Temperature (°C)	Ref.
**GAPDH/** **NM_017008.4**	F	AGATCCACAACGGATACATT	52	[67]
	R	TCCCTCAAGATTGTCAGCAA		
**LC3II/NM_199500.2**	F	GAGAAGCAGCTTCCTGTTCTGG	60	[68]
	R	GTGTCCGTTCACCAACAGGAAG		
**Beclin-1/** **NM_053739.2**	F	GAGAAGCAGCTTCCTGTTCTGG	60	[69]
	R	GTGTCCGTTCACCAACAGGAAG		
**P62/** **NM_175843.5**	F	TGCCCAGACTACGACTTGTG	56	[68]
	R	AGTGTCCGTGTTTCACCTTCC		
**ATG5/** **NM_001014250.2**	F	GCAGATGGACAGTTGCACACAC	56	[70]
	R	GAGGTGTTTCCAACATTGGCTCA		
**ATG7/** **NM_001012097.1**	F	CGTTGCCCACAGCATCATCTTC	56	[70]
	R	TCCCATGCCTCCTTTCTGGTTC		
GAPDH, glyceraldehyde-3-phosphate dehydrogenase; LC3II, microtubule-associated protein light chain 3II; P62, ubiquitin-binding protein p62; ATG5, autophagy-related protein 5; ATG7, autophagy-related protein 7				

**Table 2:** The composition of some constituents of the Purslan ethanolic extract (PEE) and Chicory water extract (CWE).

Phytochemicals	Concentration	
	PEE	CWE
**Yield (g%)**	15.72 ± 0.72	9.36 ± 0.50
**Total phenolics** **(mg catechin Eq/g extract)**	49.642 ± 0.910	16.428 ± 0.793
**Total flavonoids** **(mg gallic acid Eq/g extract)**	113.285 ± 3.011	105.071 ± 1.105
**HPLC analysis of the phenolic compounds (μg/g extract)**		
**Pyrogallol**	ND	ND
**Quinol**	ND	12.50323
**Gallic acid**	**27.68568**	9.87210
**Catechol**	**67.76362**	ND
***p*** **-Hydroxybenzoic acid**	**180.47324**	4.27578
**Catechin**	ND	0.00000
**Chlorogenic acid**	**39.94523**	8.35102
**Vanillic acid**	ND	0.00000
**Caffeic acid**	14.27399	4.48349
**Syringic acid**	**56.37858**	9.67627
***p*** **-Coumaric acid**	**31.63395**	0.00000
**Benzoic acid**	**394.93195**	ND
**Ferulic acid**	13.97240	2.27579
**Rutin**	ND	0.00000
**Ellagic**	ND	ND
**o-Coumaric acid**	18.32819	25.75831
**Resveratrol**	ND	**941.10714**
**Cinnamic acid**	ND	ND
**Quercetin**	**101.22068**	ND
**Rosmarinic acid**	**72.48203**	ND
**Naringin**	ND	**333.97923**
**Myricetin**	**112.91257**	ND
**Kaempferol**	ND	26.01053
**Total**	**1132.00209**	**1378.29289**
**Minerals (µg/g extract)**		
**Ca**	895089.527	104208.430
**Cu**	1975.3378	967.96511
**Fe**	108850.8108	31512.7095
**K**	205133.2432	1015929.7674
**Mg**	901662.2297	32384.18605
**Mn**	887.7027	577.2674
**Na**	2009375.5405	220631.2209
**P**	649056.4189	146762.7325
**Se**	45.2027	22.1512
**Zn**	4350.6081	933.9535
**Vitamins (value/g extract)**		
**Folic acid (µg)**	56.99	68.98
**Vitamin C (µg)**	40.05	63.25
**Vitamin A (IU)**	55.41	5.91
Results are presented as the mean ± SD (*n* = 3). Eq—Equivalent; ND—Not detected.		

**Table 3:** The combination index (CI) values for the PEE and CWE mixture on the tested parameters.

Parameters		CI	Effect
**In vitro antioxidant assays**			
DPPH (mg/mL)		0.730± 0.001	**Synergistic**
NO scavenging (mg/mL)		0.249± 0.020	**Synergistic**
Superoxide radical scavenging (mg/mL)		0.337± 0.017	**Synergistic**
T. antioxidant (mg/mL)		0.341± 0.022	**Synergistic**
**Seminal quality and oxidative stress markers**			
Sperm count (10^6^/mL)		1.076 ± 0.006	**Additive**
Morphology index (%)		0.985 ± 0.002	**Synergistic**
Motility type (%)	Progressive	0.981 ± 0.011	**Synergistic**
	Non-progressive	0.959 ± 0.015	**Synergistic**
Motility (%)	1st h	0.919 ± 0.013	**Synergistic**
	2nd h	0.911 ± 0.003	**Synergistic**
	3rd h	0.000 ± 0.000	**Synergistic**
Serum fructose level (mg/dL)		1.064 ± 0.011	**Additive**
Seminal fructose level (mg/g tissue)		1.019 ± 0.009	**Additive**
α-Mannosidase activity (U/mg protein)		0.123 ± 0.002	**Synergistic**
FSH (μIU/mL)		0.890 ± 0.001	**Synergistic**
LH (μIU/mL)		0.837 ± 0.004	**Synergistic**
Testosterone (ng/dL)		0.618 ± 0.011	**Synergistic**
MDA (μmol/mg protein)		0.748 ± 0.041	**Synergistic**
GSH (mM/mg protein)		0.763 ± 0.012	**Synergistic**
GPx activity (U/mg protein)		0.520 ± 0.011	**Synergistic**
GST activity (U/mg protein)		0.543 ± 0.039	**Synergistic**
**Insulin resistance markers**			
FBS (mg/dL)		0.951 ± 0.021	**Synergistic**
Insulin (𝛍𝐔/mL)		0.983 ± 0.011	**Synergistic**
HOMA-IR		0.755 ± 0.019	**Synergistic**
HOMA-β		1.227 ± 0.028	**Antagonistic**
**ER stress and autophagy parameters**			
GRP78 (Protein expression fold)		0.580 ± 0.018	**Synergistic**
Sigma 1R (ng/mg protein)		0.898 ± 0.011	**Synergistic**
PI3KC3 (ng/mg protein)		1.076 ± 0.018	**Additive**
Beclin-1	ng/mg protein	0.898 ± 0.005	**Synergistic**
	mRNA expression fold	0.557 ± 0.011	**Synergistic**
LC3II	Protein expression fold	0.543 ± 0.016	**Synergistic**
	mRNA expression fold	0.332 ± 0.022	**Synergistic**
ATG5 (mRNA expression fold)		0.384 ± 0.008	**Synergistic**
ATG7 (mRNA expression fold)		0.271 ± 0.027	**Synergistic**
P62	Protein expression fold	0.485 ± 0.148	**Synergistic**
	mRNA expression fold	0.595 ± 0.016	**Synergistic**
P-mTOR Ser2448 (Protein expression fold)		0.434 ± 0.018	**Synergistic**
CI < 1, =1, and >1 indicate synergistic, additive, and antagonistic effects, respectively.			

**Table 4:** The effects of different treatments on sperm count, motility and morphology index, fructose, FSH, LH and testosterone levels, mannosidase activity, and oxidative stress markers.

		**Control**	**PEE**	**CWE**	**PEE/CWE**	**Dexa**	**Dexa + PEE**	**Dexa + CWE**	**Dexa + PEE/CWE**
**Sperm Count** **(10^6^/^mL^)**		178 ± 8.83	189 ± 5.66 *	163 ± 8.66	169 ± 3.41	206 ± 4.24 *	168 ± 7.07 ^#,a^	185 ± 5.55 ^#,a^	200 ± 1.14 *^,#^
**Morphology Index** **(%)**		90.0 ± 5.0	92.5 ± 3.5	90.0 ± 0.0	82.5 ± 4.04	75.0 ± 0.0 *	90.9 ± 2.35 ^#^	85.6 ± 7.07 ^#^	85.5 ± 3.5 ^#^
**Motility Type** **(%)**	Progressive	86.65 ± 4.71	90.00 ± 0.00	85.00 ± 4.08	90.00 ± 0.00	47.50 ± 6.45 *	85.00 ± 1.07 ^#,a^	90.75 ± 2.50 ^#^	93.32 ± 2.35 *^,#^
	Non-progressive	13.32 ± 4.01	10.00± 0.00	15.00 ± 4.08	10.00 ± 0.00	52.54 ± 6.45 *	15.00 ± 2.07 ^#,a^	10.00 ± 1.08 ^#,a^	6.65 ± 2.05 *^,#^
**Motility** **(%)**	1st h	92.0 ± 4.5	90.0 ± 0.00	85.0 ± 7.07	90.0 ± 0.00	45.0 ± 5.77 *	87.0 ± 10.61 ^#^	93.3 ± 2.35 ^#,a^	87.5 ± 2.53 ^#^
	2nd h	65.0 ± 8.14	67.5 ± 7.68	50.5 ± 9.85	53.1 ± 7.07	11.7 ± 2.07 *	42.5 ± 10.6 *^,#^	75.5 ± 13.43 ^#^	72.1 ± 10.60 ^#^
	3rd h	5.0 ± 0.5	5.0 ± 0.0	5.0 ± 0.0	4.5 ± 0.4	0.0 + 0.0 *	5.0 ± 0.0 ^#^	5.1 ± 0.0 ^#^	5.5 ± 0.0 ^#^
**Serum Fructose level** **(mg/dL)**		124.34 ± 10.15	145.57 ± 13.96	148.67 ± 4.34*	150.88 ± 8.75 *	85.13 ± 8.799 *	136.73 ± 6.68 ^#^	133.19 ± 10.35 ^#^	143.36 ± 5.01 *^,#^
**Seminal Fructose level** **(mg/g tissue)**		148.23± 8.59	150.50 ± 8.28	156.08 ± 6.78	163.71 ± 8.23	115.79 ± 7.26 *	145.13 ± 4.09 ^#^	139.38 ± 3.64 ^#^	143.36 ± 4.90 ^#^
**α-Mannosidase activity** **(U/mg protein)**		0.18 ± 0.011	0.17 ± 0.017	0.18 ± 0.014	0.18 ± 0.018	0.13 ± 0.012 *	0.16 ± 0.016 ^#^	0.15 ± 0.030 ^#^	0.17 ± 0.016 ^#^
**FSH** **(μIU/mL)**		49.5 ± 1.80	48.4 ± 1.71	61.6 ± 4.04 *	67.65 ± 2.93 *	11 ± 1.41 *	43.9 ± 3.88 ^#,a^	53.1 ± 2.34 ^#^	50.8 ± 0.93 ^#^
**LH** **(μIU/mL)**		37.17 ± 2.02	38.5 ± 0.99	45.03 ± 2.88 *	44.425 ± 4.49 *	13.67± 2.05 *	27.47 ± 1.6 *^,#,a^	38.83 ± 1.02 *^,#^	34.2 ± 1.04 ^#^
**Testosterone** **(ng/dL)**		2.51 ± 0.30	2.19 ± 0.19	2.23 ± 0.30	2.39 ± 0.41	0.91 ± 0.10 *	1.05 ± 0.11*	1.29 ± 0.25 *	1.51 ± 0.17 *^,#^
**MDA** **(μmol/mg protein)**		0.883 ± 0.19	0.819 ± 0.06	0.654 ± 0.24	0.325 ± 0.05 *	3.064 ± 0.42 *	0.821 ± 0.12 ^#^	0.703 ± 0.15 ^#^	0.726 ± 0.11 ^#^
**GSH** **(mM/mg protein)**		3.83 ± 0.20	3.79 ± 0.18	3.71 ± 0.32	3.99± 0.44	2.11± 0.18 *	3.31 ± 0.25 *^,#^	3.28 ± 0.13 *^,#,a^	3.70 ± 0.18 ^#^
**GPx activity** **(U/mg protein)**		4.61 ± 0.31	4.09 ± 0.30	4.38 ± 0.29	4.91 ± 0.24	1.27 ± 0.21 *	3.6 ± 0.24 *^,#^	3.7 ± 0.2 *^,#^	3.4 ± 0.17 *^,#^
**GST activity** **(U/mg protein)**		1.87 ± 0.05	1.80 ± 0.07	1.78 ± 0.06	1.85 ± 0.09	1.0 ± 0.03 *	1.66 ± 0.09 *^,#^	1.58 ± 0.07 *^,#a^	1.76 ± 0.09 ^#^

Data are expressed as mean ± SD of six rats. Two-way analysis of variance (ANOVA) was used, followed by Tukey’s post hoc test (* *p* < 0.05 vs. control group, # *p* < 0.05 vs. Dexa group, and ^a^ *p* < 0.05 vs. DEXA + PEE + CWE group).

## Data Availability

All of the data is contained within the article.
